# A family cluster of schistosomiasis acquired in Solenzara River, Corsica (France) — Solenzara River is clearly a transmission site for schistosomiasis in Corsica

**DOI:** 10.1007/s00436-022-07574-9

**Published:** 2022-06-18

**Authors:** Nele Wellinghausen, Hélène Moné, Gabriel Mouahid, Axel Nebel, Dennis Tappe, Martin Gabriel

**Affiliations:** 1 MVZ Labor Ravensburg, Elisabethenstr. 11, 88212 Ravensburg, Germany; 2 UMR 5244 IHPE Interactions Hôtes-Pathogènes-Environnements, Université de Montpellier, CNRS, IFREMER, Université de Perpignan, 66860 Perpignan, France; 3 Ravensburg, Germany; 4 National Reference Centre for Tropical Pathogens, Bernhard Nocht Institute for Tropical Medicine, Hamburg, Germany

**Keywords:** Schistosomiasis, Corsica, France, Solenzara River, *Schistosoma*

## Abstract

We report a patient with urogenital schistosomiasis and three cases of subclinical infection within one family acquired from Solenzara River, Corsica, in 2019. Our cases confirm that transmission of schistosomiasis in Corsica is ongoing and has been extended from the Cavu River to the Solenzara River. Solenzara River is clearly a transmission site for schistosomiasis in Corsica. Public health efforts are recommended to uncover and prevent further cases.

## Introduction

Urogenital schistosomiasis is an emerging disease in Europe. In 2014, a first case of *Schistosoma haematobium* infection in a German boy who acquired infection in 2013 in the Cavu River in Southern Corsica (France) has been reported (Holtfreter et al. 2014), closely followed by another case in a French girl acquired in the same river (Berry et al. 2014). Surveillance studies later revealed 106 documented cases associated with the Cavu River (Noel et al. 2018). From 2015 to 2018 also, sporadic infections acquired from the Cavu River have been recorded (Ramalli et al. 2018). Recently, a first case of urogenital schistosomiasis transmitted in 2019 from a different river in Corsica, likely but not surely, the Solenzara River, has been published in a German traveller (Rothe et al. 2021). Here, we report a family cluster of schistosomiasis clearly linked to the Solenzara River in the same year.

## Case report

**Fig. 1:**
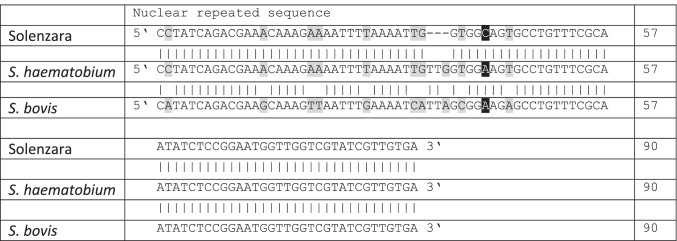
Comparison of the index patient’s nuclear repeated sequence with both *S. haematobium* and *S. bovis.* The bases highlighted in grey show the differences between the Solenzara sequence and that of *S. bovis*; the base highlighted in black is specific to the Solenzara sequence. *S. haematobium* nuclear repeated sequence (DQ157698.1: Hamburger et al. 2001). *S. bovis* nuclear repeated sequence (DQ831697.1: Abbasi et al. 2007). Three bases are lacking in the Solenzara sequence at positions 35, 36 and 37; we did not take into account these missing bases in the comparison

A 19-year-old formerly healthy young man presented to his urologist in July 2021 with macrohematuria for 2–3 weeks. Ultrasound and cystoscopy revealed a tumor of 1.5 to 2 cm in diameter on the left upper bladder wall protruding into the bladder cavity. The tumor was completely resected by transurethral resectomy, and histology showed pronounced granulomatous inflammation and suspicion of egg fragments suggestive of schistosomiasis. Urine microscopy revealed eggs with typical morphology of *S. haematobium*. In addition, elevated *Schistosoma*-specific IgG antibodies were detected in serum by enzyme-linked immunoassay (ELISA) with an index of 60 (cutoff index < 9, DRG Diagnostics, Springfield, NJ, USA) and by indirect immunofluorescence test (IIFT) with a titer > 1:160 (cutoff titer < 1:20, Euroimmun, Lübeck, Germany). The patient was treated with praziquantel for 3 days and fully recovered. Shortly after therapy, polymerase chain reaction targeting a nuclear repeated sequence was performed on the patient serum; for methods see Abbasi et al. (2007), Hamburger et al. (2001), and Moné et al. (2015). The alignment of the obtained, albeit short, sequence of the patient (90 bp) with those of *S. haematobium* and *S. bovis* was made using the BLAST tool in NCBI (Fig. 1). The percentages of similarity were 98.9% with *S. haematobium* and 88.5% with *S. bovis* sequences. Further characterization of the Schistosoma DNA by next-generation sequencing failed due to the low amount of parasite DNA detected. Microscopy and PCR results thus confirmed that the *Schistosoma* sample from the patient corresponds to *S. haematobium*. This result is concordant with the ones recovered in Corsica where both *S. haematobium* and hybrids between *S. haematobium* and *S. bovis* were found (Moné et al. 2015;Savassi et al. 2020).

The patient had never travelled to any region endemic for schistosomiasis apart from a family holiday in Corsica in the last week of August and the first week of September 2019. Since his parents were aware of the risk of schistosomiasis in Corsica, they actively avoided the Cavu River during their holidays and only bathed twice in the Solenzara River near a campsite restaurant.

After establishment of the diagnosis, his parents and 18-year-old sister were screened for *Schistosoma* infection by urinalysis, bladder ultrasound, *Schistosoma* serology, and PCR in serum. No hematuria or ultrasound abnormalities were detected, but *Schistosoma* infection was confirmed in all three family members by elevated *Schistosoma*-specific IgG antibodies, albeit the mother only showed a borderline antibody titer (Table 1). Detection of eggs in a mid-day urine sample and *Schistosoma* DNA in serum failed in all the three patients. All the family members had not been in any other country with known endemicity of schistosomiasis for the last 20 years.

**Table 1:** Serological and PCR results

Case/age	*Schistosoma* IgG ELISA (index)^a^	*Schistosoma mansoni* cercariae ELISA^b^	*Schistosoma* spp. IIFT (titer)^c^	*Schistosoma* spp. PCR in serum
Index, 19 years	60	Positive	> 1:160	Positive
Sister, 18 years	45	Positive	> 1:160	Negative
Mother, 51 years	10	Positive	< 1:20	Negative
Father, 51 years	48	Positive	1:160	Negative

^a^DRG Diagnostics, Springfield, NJ, USA, according to the manufacturer an index between 9 and 11 is regarded as borderline, an index > 11 as positive

^b^In-house test, Bernhard-Nocht-Institute for Tropical Medicine, Hamburg, Germany, positive results are explained due to serological cross-reactivity with *S. haematobium*

^c^IIFT: indirect immunofluorescence test, Euroimmun, Lübeck, Germany, titer < 1:20 are regarded as negative

## Discussion — ongoing endemicity of schistosomiasis in Corsica

After the first published case of schistosomiasis in 2014 and detection of several further patients in Corsica affecting the Cavu River, only sporadic cases were recorded in the following years. Recently, a first case of urogenital schistosomiasis was reported that was acquired in 2019 from the Solenzara River situated north of the Cavu River (Rothe et al. 2021). Interestingly, the causative *Schistosoma* species in both the Cavu and Solenzara Rivers was a hybrid between *Schistosoma haematobium* and *S. bovis* (Moné et al. 2015). Phylogenetic analyses showed that this strain was previously imported from Senegal (Boissier et al. 2016) or Benin (Savassi et al. 2020). The waters of the Solenzara River do not intermingle with those of the Cavu River but the intermediate vector, *Bulinus truncatus* snails, have been detected earlier in the Solenzara River during environmental surveys (Mulero et al. 2019). Apart from contamination of the river by an infected person, animal reservoirs have been discussed as a possible source of transmission of schistosomes in Corsica (Oleaga et al. 2019). However, an animal source has not been proven yet (Oleaga et al. 2019), and transmission by an infected person appears, thus, the most likely source of transmission to the Solenzara River. Broad thermal range of schistosomes indicates that they can overwinter under temperate climates like in Corsica (Mulero et al. 2019) and also in infected humans. In addition, *Bulinus* snails can overwinter in the rivers. Infected persons may thus reinfect the rivers in spring and may lead to new cases in late summer, as observed in our family and in other published cases.

Patient history of the family reported here revealed that all four cases did only have water contact in the Solenzara River during two bathing events, and that they avoided all other rivers in Corsica. The bathing site in the Solenzara River (GPS coordinates 41°50′37.8"N 9°20′36.3"E) was situated close to a restaurant next to a campsite and corresponds to one of the possible sources of infection of the first patient published by Rothe et al. (2021). Infection of our patients occurred 1 to 2 weeks after the infection of the abovementioned patient in August/September 2019.

## Conclusion

Our cases confirm that transmission of schistosomiasis in Corsica is ongoing and has been extended from the Cavu River to the Solenzara River. The fact that all family members acquired infection after two bathing events indicates a relevant risk of infection from the Solenzara River in August/September 2019. Public health awareness should be increased and epidemiological survey investigating the actual infection risk from the Solenzara River should be initiated. In addition, residents and tourists should be informed about the possible risk of transmission of schistosomiasis from the Solenzara River and possible unknown cases should be sought.
